# COVID-19 anxiety and quality of life among Iranian nurses

**DOI:** 10.1186/s12912-021-00800-2

**Published:** 2022-01-20

**Authors:** Zohreh Mohamadzadeh Tabrizi, Fatemeh Mohammadzadeh, Arezoo Davarinia Motlagh Quchan, Narjes Bahri

**Affiliations:** 1 Faculty Member of Paramedicine School, Sabzevar University of Medical Sciences, Sabzevar, Iran; 2 Department of Epidemiology & Biostatistics, School of Health, Social Development & Health Promotion Research Center, Gonabad University of Medical Sciences, Gonabad, Iran; 3 Department of Midwifery, Faculty of Medicine, Social Determinants of Health Research Center, Gonabad University of Medical Sciences, Gonabad, Iran

**Keywords:** Anxiety, COVID-19, Quality of life, Nurse, Iran

## Abstract

**Background:**

The Coronavirus Disease 2019 (COVID-19) pandemic has exposed nurses, who are a very important group involved in the care of these patients, to many stresses that may affect their quality of life. This study aimed to determine the relationship between COVID-19 anxiety and the quality of life among Iranian nurses.

**Method:**

This online cross-sectional study enrolled 1,131 of Iranian nurses working at the time of the COVID-19 outbreak in treatment centers in different parts of Iran from April to May 2020. The convenience sampling strategy was used. Data were collected using a demographic questionnaire, the 36-Item Short-Form Health Survey (SF-36), and Corona Disease Anxiety Scale (CDAS). The stepwise multiple linear regression models were used to examine the relationships among self-reported anxiety concerning COVID-19 and SF-36 quality of life, its components, and subscales. Partial r was used as an estimate of effect size.

**Result:**

The mean SF-36 score was 65.2 (*SD*=17.6). The mean score of the mental component summary (MCS) (*M*=56.8, *SD*=22.3) was lower than the mean score of the physical component summary (PCS) (*M*=71.6, *SD*=17.5). The mean score of COVID-19 anxiety was 17.8 (*SD*=10.5). Of the participants, 378 (33.4%; 95% CI [30.7%, 36.3%]), and 152 (13.4%; 95% CI [11.5%, 15.6%]) reported moderate and severe anxiety, respectively. According to the results of stepwise multiple linear regression model, after adjusting for possible confounding variables, the SF-36 quality of life was still significantly negatively associated with COVID-19 anxiety, with a large effect size (The partial *r* = -0.515, *p* < 0.001). The relationship between the SF-36 components and COVID-19 anxiety were also significant, and moderate to large effect sizes were observed (The partial r for (PCS; COVID-19 anxiety) = -0.404; *p* < 0.001, and for (MCS; COVID-19 anxiety) = -0.521; *p* < 0.001). In addition, significant correlation coefficients for every subscale of the SF-36 were found for COVID-19 anxiety and its two components, with small to large effect sizes (The partial correlations= -0.211 to -0.524, all *p*s< 0.001).

**Conclusions:**

The results showed that higher COVID-19 anxiety in nurses decreases their quality of life. In order to increase nurses’ quality of life during the COVID-19 pandemic, it is recommended to design and implement programs to reduce their COVID-19 anxiety.

## Introduction

In late December 2019, several cases of a pneumonia-like disease (with symptoms such as fever, difficulty breathing, coughing, and invasive lesions in both lungs) occurred in Wuhan, Central China, for unknown reasons. The disease is now called Coronavirus Disease 2019 (COVID-19) [1]. The virus causing the disease belongs to the coronavirus family that cause respiratory and intestinal infections in animals and humans, including the Middle East Respiratory Syndrome (MERS) and SARS [1]. Following the outbreak of the virus and its pandemic, on January 11, 2020, the World Health Organization (WHO) issued a statement declaring that the outbreak of the new coronavirus was the sixth leading cause of public health emergency worldwide, a threat not only to China but to all countries [2]. In mid-February 2020, Iran became the second focal point for the spread of the coronavirus in the world after China. As of August 5, 2021, the total number of COVID- 19 patients in Iran was 4,057,758 individuals and the total number of related deaths was 92,628 individuals [3]. The disease quickly became a global pandemic and severely strained countries’ healthcare system. Hospital beds were filled with an influx of COVID-19 patients. Nurses, as a group that should provide care for patients, have been under intense physical and mental stress since then.

The nature of nursing exposes the nurses to more occupational stress [4]. Evidence has even shown that nurses have higher occupational stresses than other health workers, including physicians. This evidence was reported even before the onset of the COVID-19 pandemic [5]. The pandemic exposes the nurses to higher occupational stress and anxiety compared to before. Erin et al. (2021) investigated psychosocial outcomes of the COVID-19 pandemic on healthcare workers in maternity services situated in Trabzon, Turkey and they reported the level of trait and state anxiety in maternity nurses during pandemic is higher than before pandemic [6]. Also, other evidence shows that the pandemic puts great psychological pressure on nurses which increases rates of anxiety, suicide, fear, and depression [7, 8]. One of the major concerns in this regard is the impact of anxiety caused by COVID-19 on nurses’ quality of life.

Currently, the concept of “quality of life” is a key and fundamental concept in human life. Over time, and with the improvement of health and well-being of human societies, people shifted their attention from longevity and treatment to subjective and objective issues of welfare and quality of life [9]. Therefore, quality of life has been one of the most studied topics in clinical research over the past two decades [10]. The WHO defines quality of life as the individual’s perception of their position in life in the context of the culture and value systems in which they live and in relation to their goals, expectations, standards, and priorities [10] Quality of life includes all aspects of life and is not limited to health. Occupation is a factor influencing the quality of life [11].

Nursing, as one of the first four stressful professions in the world, exposes individuals to various physical, psychological, and social stressors and threats, and undermines health and well-being [12]. Among the factors influencing nurses’ anxiety is sudden changes in patient status, frequent contact with patients’ sufferings, shift work and night shifts, the uncertainty of treatment, heavy workload, mandatory overtime, job insecurity, different working environments, entering a new working environment, difficulties of nursing profession, conflicts with physicians, conflicts with colleagues, high working hours, low income, lack of commitment of the manager or supervisor, discrimination between employees, lack of proper facilities and adequate medical equipment, non-standard and inappropriate and physical activity conditions, and disregard for the dignity and position of nurses in society [13]. These pressures can increase nurses’ anxiety and cause significant damage to their health and quality of life [14].

Since occupational conditions and stress have serious impacts on individual’s health and quality of life, addressing nurses’ health and quality of life as well as keeping them healthy, first as humans and then as people who protect the health of other community members, are particularly important [12].

The quality of life and anxiety changes in the nursing profession, can lead to significant problems in individual, social and occupational dimensions—influencing daily personal functions, such as eating, sleeping and health [15]. Therefore, this study aimed to determine the relationship between COVID-19 and Iranian nurses’ quality of life.

## Methods

### Study design, sample size, and participants

This online cross-sectional study enrolled 1,131 Iranian nurses from April to May 2020. To estimate the status (mean score) of quality of life and anxiety during the COVID-19 pandemic in Iranian nurses, the sample size based on the formula of ${\left(\frac{Z_{1-\alpha /2}+{Z}_{1-\beta }}{E}\right)}^2$, a 95% confidence interval, a test power of 0.9, and a small effect size of E=0.1 according to Cohen’s guidelines [16] was determined as 1,050. It was increased to 1,150 when a possible attrition rate of 10% was considered. The sample size was large enough for regression analysis, based on the rule of thumb (n≥ 50 + 8×the number of predictors) [17]. The study was performed using the convenience sampling strategy and an online questionnaire to prevent the spread of COVID-19. We sent a link to the online questionnaire and its relevant details via text message, related groups, and channels on the popular smartphone apps WhatsApp, Telegram, and Instagram to the Iranian nurses we knew and were easy to contact or to reach. They were asked to complete it and forward and share it with other individuals, groups, and channels of Iranian nurses they knew. The following were the inclusion criteria: (1) Iranian nationality; (2) working in the fields of nursing, anesthesiology, or the operating room in Iran during the COVID-19 pandemic; and 2) willingness to participate in the study. Participants who did not qualify and/or complete the questionnaires were excluded from the study.

### Data instruments

#### a- Demographic information questionnaire

The demographic information questionnaire included several questions about age, gender, field of study, marital status, educational level, income, work experience, smoking, exposure to COVID-19 patients, underlying diseases (including hypertension, diabetes, heart disease, high cholesterol, hyperthyroidism, hypothyroidism, kidney failure, and other), history of mental illness, and physical exercise.

#### b- Corona Disease Anxiety Scale (CDAS)

In this study, the CDAS was used to measure the anxiety levels during the COVID-19 pandemic amongst participants of the study. The scale was first developed and validated by Alipour et al. (2020) at the start of the COVID-19 pandemic in Iran. The validation of the scale was performed through different types of validity, including content validity, construct validity, convergent validity, and criterion validity. The CDAS criterion validity was examined by evaluating the degree of correlation between the CDAS and the GHQ-28 questionnaire, the findings of which indicated that the CDAS was significantly correlated with the total GHQ-28 scores (*r*=0.483) and its subscales, including anxiety (*r*=0.507), physical symptoms (*r*=0.418), social dysfunction (*r*=0.333), and depression (*r*=0.269) (All P-values less than 0.01). The CDAS has 18 items and two components. The first component (component A) includes items 1-9 that measure psychological symptoms (for example, “It makes me anxious to think about COVID-19.“ or “I feel tense when I think about the COVID-19 threat.“). The second component (component B) includes items 10-18 that measure physical symptoms (e.g., “Thinking about COVID-19 has disrupted my sleep”). The items are scored based on a 4-point Likert scale (Never=0, Sometimes=1, Often=2, Always=3). Each subscale’s total score is the sum of all related items, ranging from 0 to 27. The scale’s total score is the sum of the scores on two subscales, ranging from 0 to 54. Higher scores indicate a higher level of anxiety. Based on the scale developers’ recommended cut offs, the CDAS scores are categorized into mild (0-16 for the total score, 0-1 for physical symptoms, 0-5 for psychological symptoms), moderate (17-29 for the total score, 2-9 for physical symptoms, 6-19 for psychological symptoms), and severe (30-54 for the total score, 10-27 for physical symptoms, 20-27 for psychological symptoms). The reliability of the CDAS was confirmed with Cronbach’s alpha of 0.919 for the entire scale and 0.879 and 0.861 for the subscales of psychological symptoms and physical symptoms, respectively [18]. The reliability of the CDAS in the present study was confirmed using Cronbach‘s alpha, which the values were 0.92, 0.89, and 0.94 for the psychological symptoms, physical symptoms, and the overall scale, respectively.

#### c- 36-Item Short-Form Health Survey (SF-36)

In the present study, quality of life was assessed using the SF-36 questionnaire. The SF-36 is a self-administered general instrument that is widely used to evaluate health-related quality of life. It has 36 items in eight subscales categorizing into two generic components: physical component summary (PCS) and mental component summary (MCS). Physical component summary consists of the subscales of physical functioning (10 items), role-physical (4 items), bodily pain (2 items), and general health perceptions (5 items). Mental component summary consists of subscales of social functioning (2 items), role-emotional (3 items), mental health (5 items), and vitality (4 items). One item also individually examines changes in health. Scores are coded, summed, and converted to a scale of 0 to 100, with 0 reporting the worst and 100 reporting the best condition. Montazeri et al. confirmed the validity of the SF-36 questionnaire in a healthy population of over 15 years old in Iran (Tehran) in 2006. Cronbach’s alpha coefficients ranging from 0.77 to 0.90 for subscales and the overall scale confirmed the questionnaire reliability [19]. The reliability of the SF-36 in the present study was assessed using the Cronbach‘s alpha which was 0.92.

### Ethical considerations

The project objectives were explained at the beginning of the questionnaire so that nurses could fill in and send the questionnaires back along with the informed consent online. The participants were assured that all the information on the form would remain confidential and the results would be expressed in general. The study protocol was approved at the Research Ethics Committee of Gonabad University of Medical Sciences (Code: IR.GMU.REC.1399.008).

### Statistical analysis

Data were analyzed using the SPSS-16 software. Mean and standard deviation (SD) was used to provide descriptive statistics on quantitative variables and number (percentage) for qualitative variables. The normality for quantitative variables was examined using the Kolmogorov–Smirnov (K–S) test and skewness and kurtosis values. For all the quantitative variables, the absolute values of skewness and kurtosis were less than 2 and 7, respectively, and no severe violation of the normality assumption was found [20].

We conducted the univariate analyses to determine the relationships between the individual characteristics and the quality-of-life score. The independent-samples t-test was used to compare the mean scores of SF_36 quality of life and its components (PCS and MCS) between subgroups of individuals based on the variables of gender, marital status, educational level, income, work experience, smoking, physical exercise, underlying disease, history of mental disorders, and exposure to COVID-19 patients. Pearson’s correlation coefficients were also used to examine the relationships among self-reported anxiety concerning COVID-19 and SF-36 quality of life, its components, and subscales. Stepwise multiple linear regression analyses were used to investigate the association between COVID-19 anxiety and SF-36 quality of life, its components, and subscales in the presence of possible confounding variables, including age, gender, educational level, marital status, income, underlying medical condition, history of mental illness, exposure to COVID-19 patients, smoking, and physical exercise. Partial (adjusted) correlation coefficients were used to measure the effect size. Cohen’s recommendations were used to interpret the effect size (small = 0.1 to 0.3, medium = 0.3 to 0.5, and large = 0.5 to 1.0) [21]. For linear regression models, the K–S test and absolute values of skewness and kurtosis were used to assess the normality of residuals. Homoscedasticity, independence of errors, and multicollinearity were also examined using the scatter plot of the standardized residuals versus the standardized predicted values, the time series plot of the residuals, and variance inflammation factor (VIF), respectively. The significance level was considered less than 0.05.

## Results

### Individual characteristics

Data from 1,131 fully completed questionnaires by Iranian nurses were analyzed. The mean age of the participants was 31.3 (*SD*=7.0), ranging from 20 to 56 years. Their mean work experience was 7.7 (*SD*=6.4) years. Other characteristics of the participants are presented in Table 1.

**Table 1:** Characteristics of the participants

Variable	
**Gender, n (%)**	
Female	851 (75.2)
Male	280 (24.8)
**Educational level, n (%)**	
Associate’s degree/ Bachelor’s degree	1,029 (91.0)
Master’s degree/ PhD	102 (9.0)
**Marital status, n (%)**	
Married	719 (636.)
Single/ divorced/ widowed	412 (36.4)
**Income, n (%)**	
Low	413 (36.5)
Moderate and higher	718 (63.5)
**Field of study, n (%)**	
Nursing	862 (76.2)
Anesthesiology	145 (12.8)
Surgical technologist	124 (11.0)
**Exposure to COVID-19 patients, n (%)**	
Yes	969 (85.7)
No	162 (14.3)
**History of mental illness, n (%)**	
Yes	88 (7.8)
No	1,043 (92.2)
**Underlying medical condition, n (%)**	
** Yes**	362 (32.0)
No	769 (68.0)
**Smoking, n (%)**	
Yes	238 (21.0)
No	893 (79.0)
**Physical exercise, n (%)**	
Yes	388 (34.3)
No	743 (65.7)

*SD* Standard deviation

### COVID-19 anxiety

As is shown in Table 2, the mean scores of COVID-19 anxiety (CDAS total score) was 17.8(*SD*=10.5). Of the participants, 601 (53.2%; 95% confidence interval (CI) [50.2%, 56.1%]) of the participants had mild anxiety, 378 (33.4%; 95% CI [30.7%, 36.3%]) had moderate anxiety and 152 (13.4%; 95% CI: [11.5%, 15.6%]) had severe anxiety. Psychological symptoms related to COVID-19 had a mean score of 12.8 (*SD*=5.7), with 397 (35.1%; 95% CI [32.3%, 37.9%]), 533 (47.1%; 95% CI [44.2%, 50.1%]), and 201 (17.8%; 95% CI [15.6%, 20.1%]) of individuals having mild, moderate, and severe psychological symptoms, respectively. The mean physical symptoms score regarding COVID-19 was 4.9 (*SD*=5.5), with 102 (9.0%; 95% CI [7.4%, 10.8%]), 880 (77.8%; 95% CI [75.3%, 80.2%]), and 149 (13.2%; 95% CI [11.3%, 15.3%]), of individuals having mild, moderate, and severe physical symptoms, respectively.

**Table 2:** SF-36 quality of life, and COVID-19 anxiety scores among Iranian nurses (*n*=1131)

Variable	Mean *(SD)*
**SF-36 quality of life**	
SF-36 total score	65.2 (17.6)
**SF-36 components**	
PCS	71.6 (17.5)
MCS	56.8 (22.3)
**SF-36 subscales**	
Physical functioning	81.0(19.3)
Role-physical	60.9(35.9)
Bodily pain	70.6(24.6)
General health perceptions	61.9 (19.1)
Vitality	54.4(22.0)
Social functioning	57.1(26.7)
Role-emotional	57.5(41.7)
Mental health	58.1(21.1)
**COVID-19 anxiety**	
CDAS total score	17.7(10.5)
**CDAS components**	
Physical symptoms	4.9(5.5)
Psychological symptoms	12.7(5.7)

*CDAS* Corona disease anxiety scale; *SF-36* Short-form health survey; *PCS* physical component summary; *MCS* mental component summary

### SF- 36 Quality of life

The mean SF-36 score was 65.2 (*SD*=17.6). The mean score of MCS (*M*=56.8, *SD*=22.3) was lower than the mean score of the PCS (*M*=71.6, *SD*=17.5). Among the MCS subscales, the lowest mean score was related to vitality (*M*=54.4, *SD*=22.0) followed by social functioning (M=57.1, SD=26.7), role-emotional (*M*=57.5, *SD*=41.7), and mental health (*M*=58.1, *SD*=21.1). Among the PCS subscales, most scores were related to physical functioning (*M*=81.0, *SD*=19.3), bodily pain (*M*=70.6, *SD*=24.6), general health (*M*=61.9, *SD*=19.1), and role-physical (*M*=60.9, *SD*=35.9), respectively (Table 2).

Based on the results of univariate analyses, SF-36 quality of life and its two components’ scores were statistically significantly lower in nurses with female gender, lower-income, exposure to COVID-19 patients, history of mental illness, and underlying medical conditions (All *p*s < 0.001). Higher scores of SF-36 quality of life and its two components were observed among nurses who had physical exercise (*p*s < 0.001). Married nurses reported higher scores of the MCS component (*p*=0.008). Lower scores were observed among smoker nurses than non-smokers (*p* = 0.011). There were no significant differences among nurses with different educational levels in SF-36 quality of life or its components (*p*s > 0.05) (Table 3).

**Table 3:** SF-36 and its two components’ scores by characteristics of the participants (1131 Iranian nurses)

Variable	PCS		MCS		SF-36	
	Mean ( *SD*)	*P*-value†	Mean ( *SD*)	*P*-value†	Mean (*SD*)	*P*-value†
**Gender**		**<0.001**		**<0.001**		**<0.001**
Female	69.5 (17.9)		55.0 (22.3)		63.3 (17.7)	
Male	78.2 (14.4)		62.3 (21.4)		71.2 (15.8)	
**Educational level**		0.160		0.244		0.156
Associate’s/Bachelor’s degree	71.4(17.4)		56.5 (22.3)		65.0 (17.5)	
Master’s degree/ PhD	74.0(18.6)		59.2 (22.4)		67.6 (18.4)	
**Marital status**		0.085		**0.008**		0.710
Married	71.0 (18.2)		58.1 (22.2)		65.4 (18.0)	
Single/ divorced/ widowed	72.8 (16.2)		54.5 (22.4)		65.0 (16.8)	
**Income**		**<0.001**		**<0.001**		**<0.001**
Low	68.0 (18.7)		51.5 (22.6)		60.9 (18.0)	
Moderate and higher	73.7 (16.5)		59.8 (21.6)		67.7 (16.8)	
**Exposure to COVID-19 patients**		**0.001**		**0.001**		**<0.001**
Yes	70.9 (17.5)		55.9 (22.3)		64.4 (17.5)	
No	75.8 (17.2)		62.2 (21.9)		70.0 (17.3)	
**History of mental illness**		**<0.001**		**<0.001**		**<0.001**
Yes	60.4 (17.7)		41.5 (17.2)		52.4 (15.4)	
No	72.6 (17.2)		58.1 (22.2)		66.3 (17.3)	
**Underlying medical conditions**		**<0.001**		**<0.001**		**<0.001**
Yes	65.1 (18.0)		51.7 (22.2)		68.0 (16.8)	
No	74.7 (16.4)		59.2 (21.8)		59.3 (17.8)	
**Smoking**		0.910		**0.011**		0.154
Yes	71.5 (16.8)		53.5 (22.0)		63.8 (17.3)	
No	71.7 (17.7)		57.7 (22.3)		65.6 (17.7)	
**Physical exercise**		**<0.001**		**<0.001**		**<0.001**
Yes	74.0 (16.3)		60.1 (21.5)		68.0 (16.5)	
No	67.1 (18.9)		50.5 (22.6)		60.0 (18.4)	

*PCS* Physical component summary; *MSC* Mental component summary; *SD* Standard deviation; † Independent-samples t-test

### The relationships between SF-36 quality of life and COVID-19 anxiety

Based on the results of bivariate, unadjusted Pearson correlation coefficients the SF-36 quality of life, its two components, and all SF-36 subscales were negatively correlated with COVID-9 anxiety (CDAS total score) and its two components (Table 4).

**Table 4:** Correlation coefficients between SF-36 quality of life, its components, and subscales with COVID-19 anxiety (CDAS score and its components) among Iranian nurses (*n*=1131)

Variables	CDAS Total score		CDAS component A: Physical symptoms		CDAS component B: Psychological symptoms	
	Correlation coefficient*		Correlation coefficient*		Correlation coefficient*	
	**Raw†**	**Adjusted‡**	**Raw†**	**Adjusted‡**	**Raw†**	**Adjusted‡**
**SF-36 overall**						
SF-36 total score	**-0.561**	**-0.515**	**-0.541**	**-0.501**	**-0.509**	**-0.407**
**SF-36 components**						
PCS	**-0.461**	**-0.404**	**-0.458**	**-0.411**	**-0.404**	**-0.343**
MCS	**-0.565**	**-0.521**	**-0.529**	**-0.491**	**-0.527**	**-0.478**
**SF-36 subscales**						
Physical functioning	**-0.331**	**-0.286**	**-0.338**	**-0.300**	**-0.281**	**-0.234**
Role-physical	**-0.314**	**-0.262**	**-0.323**	**-0.278**	**-0.266**	**-0.211**
Bodily pain	**-0.388**	**-0.340**	**-0.403**	**-0.357**	**-0.325**	**-0.274**
General health perceptions	**-0.404**	**-0.359**	**-0.388**	**-0.345**	**-0.420**	**-0.369**
Vitality	**-0.498**	**-0.453**	**-0.457**	**-0.414**	**-0.474**	**-0.423**
Social functioning	**-0.524**	**-0.509**	**-0.505**	**-0.486**	**-0.474**	**-0.452**
Role-emotional	**-0.419**	**-0.391**	**-0.403**	**-0.380**	**-0.380**	**-0.350**
Mental health	**-0.489**	**-0.443**	**-0.447**	**-0.403**	**-0.467**	**-0.421**

*CDAS* Corona disease anxiety scale; *SF-36* Short-form health survey; *PCS* physical component summary; *MCS* mental component summary; † Pearson’s correlation coefficients; ‡ Partial correlation coefficients based on a stepwise multiple linear regression analysis adjusted for age, gender, educational level, marital status, income, underlying medical condition, history of mental illness, exposure to COVID-19 patients, smoking, and physical exercise; *All correlation coefficients are significant at 0.001 level

According to the results of stepwise multiple linear regression model, after adjusting for age, gender, educational level, marital status, income, underlying medical conditions, history of mental illness, exposure to COVID-19 patients, smoking, and physical exercise, the SF-36 quality of life was still significantly negatively associated with COVID-19 anxiety (Table 5), with a large effect size (The adjusted partial r: -0.515, *p* < 0.001) (Table 4). According to standardized regression coefficients, the most important factor in determining the quality of life score was COVID-19 anxiety ($\beta$=-0.47). For each unit of increase in mean anxiety score, quality of life score reduced by 0.79 units (*p* < 0.001). Also, results showed that the mean quality of life score in males was 6.52 unit higher compared to females; people with underlying diseases and a history of mental illness had 5.32 and 8.09 units lower quality of life, respectively; and people with lower income had 4.31 units lower quality of life (*p*s < 0.001). Physical activity, smoking, and exposure to COVID-19 patients had a statistically significant relationship with quality of life such that nurses in contact with COVID-19 patients ($\beta$=-0.07, *p*=0.002) or smoking nurses ($\beta$=-0.08, *p*=0.001) had a lower quality of life. In contrast, people with higher physical activity had a higher quality of life score ($\beta$=0.13, *p* < 0.001) (Table 5). The total variance of anxiety explained by the independent variables in the model was 41.4% ( (8, 1122) =100.719, *p* < 0.001, Adjusted *R*^2^= 0.414). The results of stepwise multiple linear regression models, for the relationship between the SF-36 components and COVID-19 anxiety were similar (SF-36; COVID-19 anxiety) (Table 5), and moderate to large effect sizes were observed (The partial r for (PCS; COVID-19 anxiety): -0.404 and for (MCS; COVID-19 anxiety): -0.521, *p*s < 0.001) (Table 4).

**Table 5:** Factors associated with SF-36 quality of life and its components among Iranian nurses (*n*=1131) based on the stepwise multiple linear regression

	PCS			MCS			SF-36		
**Variables**	**B (S.E)**	$\beta$	***P***	**B (S.E)**	$\beta$	***P***	**B (S.E)**	$\beta$	***P***
**Age**	-0.15 (0.06)	-0.06	**0.016**	0.33 (0.07)	0.11	**<0.001**	---	---	---
**Gender**: Male ^a^	7.20 (1.08)	0.17	**<0.001**	5.78 (1.30)	0.11	**<0.001**	6.52 (1.01)	0.16	**<0.001**
**Educational level**: Master’s degree/PhD ^b^	---		**---**	---		**---**	---		**---**
**Marital status**: Married ^c^	---		**---**	---		**---**	---		**---**
**Income**: Low ^d^	-4.10 (0.91)	-0.11	**<0.001**	-4.73 (1.10)	-0.10	**<0.001**	-4.31 (0.85)	-0.12	**<0.001**
**Underlying medical condition**: Yes ^e^	-5.32 (0.88)	-0.16	**<0.001**	-4.68 (1.16)	-0.09	**<0.001**	-5.32 (0.88)	-0.14	**<0.001**
**History of mental illness**: Yes ^e^	-6.92 (1.63)	-0.10	**<0.001**	-9.83 (1.96)	-0.12	**<0.001**	-8.09 (1.52)	-0.12	**<0.001**
**Exposure to COVID-19 patients**: Yes ^e^	-3.63 (1.24)	-0.07	**0.004**	-2.95 (1.49)	-0.05	**0.048**	-3.51 (1.15)	-0.07	**0.002**
**Smoking**: Yes ^e^	-2.58 (1.14)	-0.06	**0.024**	-4.40 (1.37)	-0.08	**0.001**	-3.43 (1.05)	-0.08	**0.001**
**Physical Exercise**: Yes ^e^	4.34 (0.91)	0.12	**<0.001**	5.82 (1.10)	0.12	**<0.001**	4.98(0.859)	0.13	**<0.001**
**COVID-19 anxiety**	-0.63 (0.04)	-0.37	**<0.001**	-1.04 (0.05)	-0.49	**<0.001**	-0.79 (0.04)	-0.47	**<0.001**
	** F(9,1121)=59.707, *P*<0.001** **Adjusted** ***R***^**2**^**= 0.319**			** F(9,1121)=82.523, *P*<0.001** **Adjusted** ***R***^**2**^**=0.394**			** F(8,1122)=100.719, *P*<0.001** **Adjusted** ***R***^**2**^**= 0.414**		

Notes: S.E, Standard Error; B: Unstandardized coefficient; $\beta$: Standardized coefficient; * *P* < 0.2; ^a^ reference category: Female; ^b^ reference category: Associate or bachelor’s degree; ^c^ reference category: Single/Widowed/Divorced; ^d^ reference category: Moderate or above; ^e^ reference category: No

In addition, significant correlation coefficients for every subscale of the SF-36 were found for COVID-19 anxiety and its two components, with small to large effect sizes (The partial correlations: -0.211 to -0.524, all *p*s < 0.001) (Table 4).

## Discussion

This cross-sectional study aimed to determine the effect of COVID-19 anxiety on the quality of life of Iranian nurses. The results showed that after adjusting the effect of individual characteristics, not only was there a statistically significant relationship between COVID-19 anxiety and quality of life, but the most important factor in determining the quality of life score was COVID-19 anxiety such that for each unit increase in mean anxiety score, quality of life score reduced by 0.81 units.

Other results indicated that 13.4% of nurses had severe COVID-19 anxiety. Hang et al. (2020) showed that COVID-19 anxiety level in nurses is higher than other occupational groups and they are more exposed to mental health issues [22]. Nemati et al. (2020) assessed nurses’ anxiety level in the face of COVID-19 and found that the level of anxiety caused by COVID-19 was high [23].

Nurses are exposed to a variety of stressors due to the nature of their work, including prolonged and continuous contact with critically ill and dying patients, extreme responsibility, excessive occupational demands from patients and their family, not enjoying welfare and recreational facilities in the society, rapid advances in technology, dealing with the reality of death and the lack of psychological support and increasing pressure of laws and regulations, all having a negative impact on nurses’ mental health [24].

Full-time work, high work pressure, and shift work leave nurses almost no time for social activities. The environmental stresses experienced by nurses can increase anxiety and depression and thus affect their quality of life [24]. Potas et al. (2021) reported similar results and their finding showed trait anxiety, psychological health, and social isolation were the main factors with statistically significant indirect effects on the quality of life of Turkish nurses [25]. Also, Korkmaz et al. (2021) reported a negative correlation between Beck Anxiety Inventory scores and the WHOQOL-BREF scores among healthcare workers, who were employed in the COVID-19 outpatient clinics [26]. Çelmeçe & Menekay (2020) reported contrast results and their finding showed that there is no significant correlation between trait anxiety and quality of life in healthcare professionals caring for COVID-19 patients [27]. Özyürek et al. (2021) reported the quality of life and anxiety have a significant difference among nurses working in a university hospital [28].

In Filali et al. study (2017) anxiety is negatively correlated with quality of life, meaning that quality of life decreases with increasing anxiety [29]. The results of other studies also showed a negative correlation between anxiety and quality of life [30, 31].

The results of this study showed that the mean scores of physical and psychological stresses were 12.8 (*SD*=5.7) and 4.9(*SD*=5.5), respectively. A study by Judaki et al. (2019) on nurses showed that psychological domain 51.77(*SD*=8.52) scored the highest while physical domain 34.14(*SD*=8.16) scored the lowest mean score of quality of life [12]. Physical factors that cause job stress include high workload, long working hours and lack of support and inability to leave work and rest, which can cause musculoskeletal injuries in nurses and cause a decline in their quality of life [19]. Also, the results of other studies that examined the effect of anxiety on the quality of life of patients with a variety of respiratory problems showed a statistically significant relationship between anxiety and quality of life [32–38]. The work environment and activities related to nurses’ work are threatening and cause anxiety, and anxiety expose nurses to harms. They also are exposed to the dangers of unhealthy lifestyles due to the nature of their occupation, which is of the major concerns of health professionals [13].

The results of the present study showed that the gender of nurses affected their quality of life as male nurses had a higher quality of life. While the results of various studies showed no statistically significant relationship between gender and quality of life [24, 39, 40], Pashib et al.(2016) showed that females have a higher quality of life than males, which is not consistent with the present study [41]. Whereas, Nasiri Qabaei et al. (2016) showed that males have a better quality of life than females, which is consistent with this study. Regarding the lower quality of life in female nurses, we can point to their numerous roles, which imposes several responsibilities on them in daily life. In addition to their job responsibilities, female nurses have to take care of their personal and family affairs throughout the day [19], as well as other roles such as childcare, which altogether deplete their energy and affect their quality of life [42].

Other results in the present study showed that nurses with low incomes had lower a quality of life. Shafi’pour et al. (2016) also showed that income adequacy affects the quality of working life [39], while Moqarrab et al. (2013) showed that monthly income does not affect the quality of working life [40]. Another study found that employees with higher incomes found themselves more successful and with better career prospects [43]. However, the results of previous studies are contradictory, and this may be due to differences in their target population or methodological designs. It is clear that more studies are needed to assess the relationship between income and quality of life.

In the present study, conducted at the time of the COVID-19 outbreak in Iran, information was collected using the capacity of social networks to observe the principles of health and social distancing, which is one of the strengths of the present study. Another strength of this study was sampling throughout all region of Iran. The present study had some limitations. First, the sampling strategy was non-probability convenience sampling that could cause selection bias and limit generalizability. Second, this was a cross-sectional study and could not prove the causality relationships. Third, self-reported questionnaires were used to collect data, in which, mental states of nurses could affect their answers. Individual differences of the participants could also affect their understanding of the quality of life, which suggests that other data collection methods, such as interviews, be used in future studies. Regarding the results we suggest to policy makers that developing appropriate strategies to protect nurses from COVID-19 related anxiety, which may improve their quality of life as well as quality of care.

## Conclusions

The overall results of this study revealed a significant statistical relationship between quality of life and COVID-19 anxiety such that increasing COVID-19 anxiety leads to a decrease in quality of life. Due to the inevitability of some factors that cause anxiety in nurses and the need to prevent physical, psychological and behavioral effects of anxiety on nurses, taking measures to improve working conditions and reduce nurses’ anxiety is necessary. Improving management methods in the nursing system, proper communication with nurses and their support, creating a suitable environment for nurses to continue their professional activities, establishing effective incentives, changing the management of nurses’ working hours and teaching coping methods are among the measures that managers of healthcare organizations can currently take to reduce COVID-19 anxiety.

## Data Availability

The datasets used and/or analyzed during the current study are available from the corresponding author on reasonable request.
